# Pilot Implementation of a Post–Hypertensive Disorders of Pregnancy Education and Follow-Up Package for Health Care Providers: Protocol for a Mixed Methods Pilot Study

**DOI:** 10.2196/81069

**Published:** 2026-02-10

**Authors:** Jennifer Elizabeth Green, Heike Roth, Ben Harris-Roxas, Kathleen Baird, Caroline Guirgis, Zarin Gundevia, Amanda Henry

**Affiliations:** 1 Collective for Midwifery, Child and Family Health, Faculty of Health, School of Nursing and Midwifery, University of Technology Sydney, Building 10, Level 11, 235 Jones Street, Sydney, NSW 2007, Australia; 2 School of Clinical Medicine, Faculty of Medicine & Health, UNSW Sydney, Sydney, Australia; 3 School of Population Health, Faculty of Medicine & Health, UNSW Sydney, Sydney, Australia; 4 Faculty of Health, University of Technology Sydney, Sydney, Australia; 5 Myhealth Newtown, Sydney, Australia; 6 Royal North Shore Hospital, Sydney, Australia; 7 Sutherland Hospital, Sydney, Australia; 8 Randwick Doctors Medical Centre, Sydney, Australia; 9 The George Institute for Global Health, Sydney, Australia

**Keywords:** hypertensive disorders of pregnancy, transitions of care, implementation, women's health, long-term health, cardiovascular, primary care, primary health care, health services administration, metabolic diseases

## Abstract

**Background:**

Medical complications of pregnancy provide a window into a woman’s future health risk. Hypertensive disorders of pregnancy (HDP) affect 1 in 10 pregnant women and elevate the risk for women of experiencing long-term health complications within 5 years of the affected pregnancy, continuing lifelong. These risks include a doubled to tripled risk of developing cardiovascular disease, a doubled risk of developing type 2 diabetes, and a 5- to 10-fold risk of developing chronic kidney disease. Early assessment and intervention following HDP are therefore crucial to improving women’s life-course health trajectory, as well as outcomes for any subsequent pregnancies. However, previous research has shown that Australian women and their primary health care providers are largely unaware of ongoing health risks and the necessary follow-up screening and assessments. Primary care providers also receive inadequate hospital-to-community handover and support to promote preventive health measures to women following pregnancy complications. Consequently, post-HDP care remains insufficient for optimizing long-term health.

**Objective:**

This study aims to (1) explore whether a post-HDP education and follow-up service package can be designed, developed, and implemented among targeted general practitioners (GPs) and maternity hospitals across Sydney, Australia, and (2) evaluate whether the post-HDP education and follow-up package can address knowledge gaps among health care providers regarding the long-term health risks after HDP and build capacity among GPs to implement evidence-based care.

**Methods:**

This pilot study will design, develop, and implement a post-HDP education and follow-up package (“the package”) adopting a collaborative and implementation methodological approach. The package, designed by expert health care providers and informed by prior evidence-based research, will include educational materials, improved hospital-to-community handover, and a funded 6-month postpartum visit.

**Results:**

Data collection occurred over an 18-month implementation and follow-up period between April 2024 and October 2025. Sixteen GPs across the Central and Eastern Primary Health Network (CESPHN) were recruited, along with their antenatal shared care (ANSC) affiliated tertiary referral hospitals in Sydney. Postimplementation data collection and analysis is planned for completion throughout 2026.

**Conclusions:**

Mixed methods evaluation will assess the efficacy, acceptability, and utility of the post-HDP package among health care providers and inform its suitability for deployment at scale.

## Introduction

### Background

Pregnancy-induced complications provide an opportunity to facilitate women’s engagement with primary health care services to promote health and reduce the burden of chronic disease in later life [1,2]. However, strategies for preventive interventions are lacking [3,4]. Supporting primary care services to enable this is a vital part of such health promotion endeavors [5,6]. Promoting population health and well-being throughout the life course is a critical global strategic objective, calling for an integrative, multisectoral approach [7]. Interdisciplinary postnatal care is acknowledged to influence short- and long-term health [1,8]. However, although the desirability of integrated health care is widely accepted throughout health policy, effective and sustainable implementation remains challenging [7,9,10].

Previous mixed methods Australian research [4,11-13] identified knowledge gaps of the long-term health risks following hypertensive disorders of pregnancy (HDP) among both women and their health care providers (HCPs). Among HCPs, knowledge gaps were identified in the classification, signs, and symptoms of various HDP and, specifically, the link between all HDP and increased cardiovascular disease and metabolic disorders in women and their children [4,13]. These findings cemented the necessity of addressing these gaps to improve long-term health outcomes for women following HDP [12,13].

The Society of Obstetric Medicine in Australia and New Zealand (SOMANZ) [14] has published recommendations to guide short- and long-term postpartum care after HDP. Addressing identified knowledge gaps and preferences of HCPs following HDP through a collaborative design of a post-HDP education and follow-up package (hereafter referred to as “the package”) may also contribute to improved early intervention for modifiable health risks following HDP. Addressing HCP preferences for content and delivery seeks to enhance clinical integration between tertiary maternity providers and primary health care practitioners, as well as facilitate ongoing counseling and management of health risks after HDP. This study provides a unique opportunity to translate research findings into clinical practice using implementation methodology. This evidence-based approach supports optimal health service design and improved preventive and primary health outcomes. Upon completion of this research, findings will further inform adjustments to existing resources to prepare the package for broader scale-up. This may also include insights for future development of such an approach for all pregnancies, particularly those affected by pregnancy complications with associated long-term health risks.

Through a collaborative design process, using earlier research findings, this protocol describes the design, development, and implementation of a targeted post-HDP education and follow-up package for HCPs. The TIDieR (Template for Intervention Description and Replication) and StaRI (Standards for Reporting Implementation Studies) checklist will be used to support replicability for health service quality improvement [15,16].

Separate complementary research is currently underway focused on addressing the educational preferences and follow-up needs of women after HDP [17]. However, we acknowledge that the women receiving care from participating general practitioners (GPs) and maternity care providers may also, by association, benefit from improved knowledge and follow-up care facilitated throughout the study. While not specifically assessed in this study, increased HCP knowledge has the potential to improve outcomes for women at risk and reduce the distress for a woman, her family, and the community in the event of an adverse cardiovascular event.

### Aims

This study aims to (1) explore whether a post-HDP education and follow-up service package can be designed, developed, and implemented among targeted GPs and maternity hospitals across Sydney, Australia, and (2) evaluate whether the post-HDP education and follow-up package can address knowledge gaps among health care providers regarding the long-term health risk after HDP and build capacity among GPs to implement evidence-based care.

### Objectives

The objectives of this study are to design, develop, implement, and assess the efficacy, acceptability, and utility for health care providers of an evidence-based post-HDP education and follow-up package for women in the first 12 months following a hypertensive pregnancy (Table 1).

**Table 1.** Objectives and evaluation mapping.

Objective	Tool	Data	Evaluation	Time
Primary objective(s)				
Development of package	Evidence-based research: Academic research Policy and guidelines Co-design: Steering committee consultation Identification of platforms in consultation with steering committee	Literature review [18] Prior evidence reporting on HCP^a^ knowledge gaps and education preferences regarding long-term health risks after HDP^b^ [4,19] SOMANZ^c^ guidelines [14]	Steering committee approval Process and postimplementation interview data with hospital-based health care providers to describe the design and implementation of the package	Preimplementation Context-specific iterations throughout implementation period in consultation with site champions/practices (implementation science iterations)
Assess implementation success and sustainability	Online surveys (GP^d^) Semistructured interviews (HCP and GP)	Qualitative and quantitative survey data Qualitative interview data	Aligning with CFiR^e^ and RE-AIM^f^ implementation methodology assessing fidelity and the enablers and barriers to implementation.	Collected before, mid-way, and after implementation Collected during and after implementation
Efficacy of education package	Website data HealthPathways data	Deidentified quantitative data	Analyzing website and HealthPathways access and traffic	Collected after implementation
Efficacy of follow-up package	Discharge referral communication between hospital and GP 6-month follow-up appointments with participating GP	Deidentified data relating to women receiving care from participating ANSC^g^ GP. Collected from participating GP and associated maternity service.	Quantitative analysis monitoring women discharged from maternity service with those followed up at participating GP	Collected mid-way and after implementation
Acceptability and utility of the package (GP)	Online surveys	Qualitative and quantitative survey data	Quantitative survey analysis will be undertaken using SPSS	Collected before, mid-way, and after implementation
Acceptability and utility of package (GP and other HCPs)	Semistructured interviews	Qualitative data	Thematic analysis	Collected after implementation
Secondary objective(s)				
Changes in HCP confidence in transmission of information	Online surveys Semistructured interviews	Qualitative and quantitative survey data Qualitative interview data	Comparing baseline confidence with postimplementation analysis	Collected before, mid-way, and after implementation
Changes in HCP capability to provide evidence-based follow-up	Online surveys Interviews	Qualitative and quantitative survey data Qualitative interview data	Assessing follow-up package including, discharge communication and adherence to 6-month follow-up appointment	Collected before, mid-way, and after implementation
Collection of data informing potential scale-up of the package	Online surveys Semistructured interviews	Qualitative and quantitative survey data Qualitative interview data	Quantitative survey data and qualitative free-text analysis. Thematic analysis from interview data collection.	Collected before, mid-way, and after implementation

a HCP: health care provider.

b HDP: hypertensive disorders of pregnancy.

c SOMANZ: Society of Obstetric Medicine of Australia and New Zealand.

d GP: general practitioner.

e CFiR: The Consolidated Framework for Implementation Research.

f RE-AIM: Reach, Effectiveness, Adoption, Implementation, Maintenance.

g ANSC: antenatal shared care.

## Methods

### Study Design

This study will utilize a multisite hybrid type 2 implementation study design [20,21]. A hybrid implementation study design acknowledges a dual focus to research to determine both clinical effectiveness and implementation strategies promoting accelerated research translation. This hybrid type 2 implementation study design aims to determine the effectiveness of an intervention pertaining to improved HCP knowledge and delivery of care after HDP, while also assessing the acceptability and utility of the implementation strategy (“the package”) [20,21]. Evaluation will be undertaken using mixed methods. Mixed methods evaluation is supported as an appropriate method for pragmatic research and implementation science. It facilitates a multidimensional lens to measure the drivers of implementation success and sustainability [22-24].

1. Assessment of acceptability and utility of the post-HDP package will require health care provider insight. This will be achieved quantitatively by survey data collection before, during, and after implementation, and qualitatively through semistructured interviews after implementation.
2. Assessment of the efficacy of system change lends itself to a quantitative analysis, which will be achieved by monitoring the number of women followed up by their GP throughout the study period.
3. Changes in HCP knowledge and confidence in transmitting evidence-based information to women regarding long-term health risks following HDP will be assessed quantitatively before, during, and after implementation via survey data collection.

**Figure 1.**
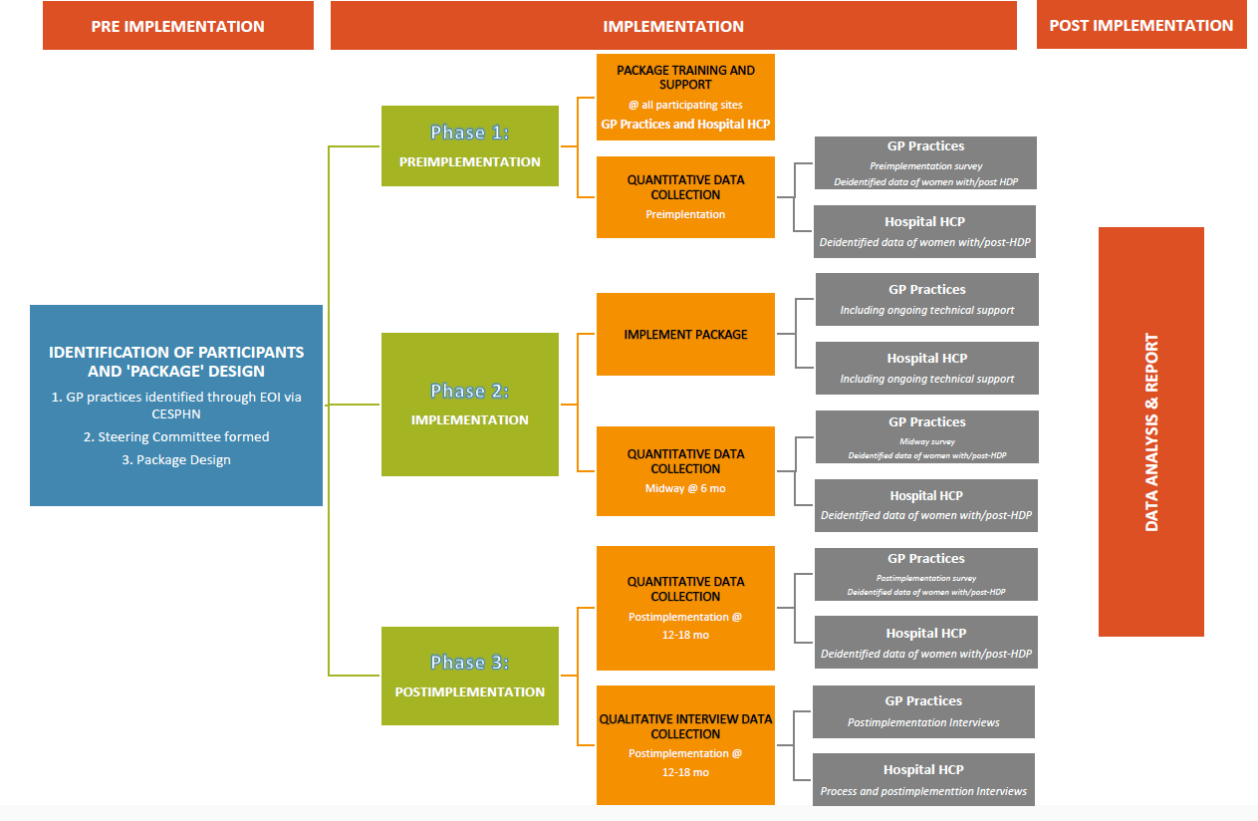
Study diagram. CESPHN: Central and Eastern Primary Health Network; EOI: expression of interest; GP: general practitioner; HCP: health care provider; HDP: hypertensive disorders of pregnancy.

The study will be conducted in 3 phases: phase 1 (preimplementation), phase 2 (implementation), and phase 3 (postimplementation) (Figure 1).

#### Phase 1: Preimplementation

The preimplementation phase includes:

- Recruitment,
- The package co-design, and
- Preimplementation survey data collection with participating GPs.

#### Phase 2: Implementation

The implementation phase includes:

- Implementation of the package at participating GP practices and affiliated hospitals over an 18-month period,
- Training and implementation support regarding the package at participating practices and hospitals, and
- Midway implementation survey data collection with participating GPs 6 months following implementation.

#### Phase 3: Postimplementation

The postimplementation phase includes:

- Postimplementation survey data collection with participating GPs at the conclusion of the implementation period and
- Semistructured qualitative interviews with health care providers (GPs and hospital-based staff) within 6 months of the implementation period concluding.

### Sample Size

The planned sample size consists of at least 10 GPs (n=10) employed in participating GP practices where implementation and evaluation of the package will occur. The planned sample size aims to provide sufficient numbers for testing the acceptability and utility of the package, and the potential for scale-up. In keeping with the pilot and implementation science nature of the study, no formal power calculation was done.

Key midwifery and obstetric staff at the linked maternity sites involved in care of women with HDP and/or GP antenatal shared care (ANSC) will also be interviewed (anticipated n=5‐10).

### Setting and Participants

The study will specifically seek to include ANSC GPs from practices within the Central Eastern Sydney Primary Health Network (CESPHN) and their affiliated maternity care providers. With a reported 19,356 births within CESPHN in 2021, CESPHN covers a large and diverse population, providing services across approximately 590 km^2^ [25]. The inclusion of ANSC GPs will promote a cohort engaged with women’s health throughout the perinatal period with already established relationships with maternity care providers. Furthermore, the diversity of the population combined with the locality of tertiary level maternity services within CESPHN is appropriate in the context of providing complex maternity care such as HDP.

### Eligibility Criteria

The following eligibility criteria will be used for this study:

- ANSC GPs within the participating CESPHN practices caring for women who experience HDP
- Maternity services within CESPHN collaborating with identified ANSC GP/GP practices participating in study providing care to women experiencing HDP

### Recruitment

Expression of Interest process for CESPHN GP practices, including recruitment of GP representatives for the package co-design and study Steering Committee.

Recruitment of affiliated hospitals via targeted emails and meetings with relevant maternity service providers.

### Ethical Considerations

Ethical approval has been obtained from the appropriate Human Research Ethics Committees across 3 hospital sites in Sydney (2023/ETH00812 | X23-0281 & 2023/STE01377 | 2023/STE01378 | 2023/STE01379). Ethical ratification was also granted by the University of Technology Sydney (ETH23-8511) and University of New South Wales.

The study will be conducted as outlined throughout the approved study protocols associated with ethical approval. Written informed consent for all aspects of the study will be obtained from GP participants and from hospital providers for their participation in interviews. Women being cared for will not provide informed consent as post-HDP follow-up is recommended standard of care [14].

### The Post-HDP Package

#### Design

The package concept was primarily informed by prior research and evidence-based practice guidelines [4,14,26-31]. To develop a feasible, appropriate, and acceptable post-HDP package for HCPs, an expert project steering committee was formed. This collaborative approach aligns with an experience-based co-design process aimed at improving knowledge and capacity to promote evidence-based integrated health care. This approach specifically addresses the needs of the HCP end users [32,33].

The project steering committee consists of the research team, site-specific hospital-based maternity care providers, GPs, and representation from the CESPHN. The multidisciplinary team reflects targeted expertise across primary care, midwifery, obstetrics, care related to HDP and post-HDP follow-up, integrated care, and research design.

The project objectives specifically outline the design, implementation, and evaluation of a post-HDP package targeted at HCPs. Therefore, women were not included in the steering committee or co-design. Separate complementary research is currently underway focused on addressing educational preferences and follow-up needs of women after HDP [17].

A steering committee–led, co-designed post-HDP package comprising the following components will be implemented.

#### Post-HDP Education Package

The education package will include:

1. A dedicated, password-protected website for HCPs providing tailored post-HDP education regarding long-term health risks.
2. A prerecorded webinar series for health care providers—made available through the dedicated website—outlining long-term health risks following HDP, recommended follow-up care, and case study examples.
3. A dedicated post-HDP “HealthPathway” [34], which is an online health information tool designed for GPs and HCPs, aims to provide context-specific health education, assessment, management, and referral information to support clinical decision-making, providing recommended education and follow-up for women following HDP.
4. Hard-copy and electronic materials provided to participating GPs to facilitate information transmission of the long-term health risks borne by women following HDP, including recommended follow-up care.

#### Post-HDP Follow-Up Package

Through site-specific specialist consultation, the package will be adapted to each site-specific needs to align with variations in local operating procedures.

1. *Post-HDP hospital discharge pathway*: Dedicated post-HDP discharge communication will include post-HDP–specific risk information and defined follow-up care.
2. *Reminder system for GPs to recall women after HDP for recommended follow-up*: A reminder system will be enabled utilizing GP practice management systems to recall women following HDP for recommended follow-up. This process will be managed by the GP practices individually, is reflected in GP practice remuneration, and will be agreed on prior to participation.
3. *Postnatal follow-up appointment at 6 months for women post HDP*: Aligning with evidence-based guidelines [14], a 6-month post-HDP visit will be offered to women by the participating GP. Costs will be covered by allocated research funds and reflected in the GP practice’s agreed remuneration to eliminate any out-of-pocket costs for the participating GP practices or women attending the visit.

### Implementation

Extensive consultation will occur across each participating site (GPs and maternity hospitals) to ensure development of a context-appropriate intervention within the constraints of local operating procedures.

This flexible approach, adopting context-specific design, aligns with a realist approach to implementation science to promote sustainable and effective implementation [22]. This further aligns with the prior research undertaken by Roth et al [4] exploring health care provider preferences regarding education on long-term health after HDP.

### Implementation Support

The researcher (JG), along with site “champions” (nominated by site-specific representatives and principal investigators), will facilitate implementation support, including the development of educational resources and quick reference guide dissemination, in-service education, and face-to-face training with staff tasked with implementing the post-HDP package.

Sustained support will continue throughout the study period. This may include, but is not limited to, adapting implementation strategies, which will be documented according to the hybrid implementation study design.

### Data Collection and Analysis

#### Quantitative Data Collection

Survey data will be collected using the REDCap (Research Electronic Data Capture) database (University of Technology Sydney, administered and secured).

Deidentified quantitative data on educational website access and general traffic will be collected for analysis to evaluate health care provider acceptability and utility. This will include the number of clicks on each page and/or their embedded items and length of time spent on each page.

Deidentified data relating to women receiving care from participating GPs with post-HDP or referred to participating GP practices will be collected from participating maternity hospitals and GP practices. These data will be sourced by reports managed by health informatics personnel, as agreed within the participating sites within the study period.

#### Qualitative Data Collection

Semistructured interviews with all participating GPs will occur within 6 months of completion of the implementation study period. Hospital HCPs participating in the study will receive an invitation to attend a postimplementation interview via email. Semistructured interviews with all consenting hospital HCPs will occur within 6 months of the completion of the study period. Interview data will be collected either in person or by telephone or videoconference, depending on participant preference, by an investigator/researcher (JEG).

#### Data Analysis

Survey data will be analyzed descriptively for each time point and comparatively across preimplementation, mid-way, and postimplementation periods. Interview data will be analyzed using thematic analysis as described by Braun and Clarke [35].

Deidentified data relating to women receiving care from participating GPs and maternity care providers will be analyzed quantitatively to determine the number of women discharged from maternity care providers and those followed up with by participating GPs. This analysis will determine the efficacy of hospital-to-GP communication.

Categorical data will be presented as numbers and proportions. Quantitative survey analyses will be undertaken using SPSS Statistics for Windows, version 29 (IBM Corp).

## Results

Data collection occurred over an 18-month implementation and follow-up period between April 2024 and October 2025. A total of 16 GPs across the CESPHN were recruited, along with their ANSC affiliated tertiary referral hospital in Sydney. Postimplementation data collection and analysis is planned for completion throughout 2026. This study will provide mixed methods data on the efficacy, acceptability, and utility of a co-designed, targeted, and evidence-based post-HDP education and follow-up package for HCPs. The results will also describe the co-design of the package, highlighting the barriers and enablers to effective implementation with a view to informing broader future scale-up.

## Discussion

### Principal Purpose

Given the strong association between HDP and increased risk of chronic disease [30,36-39], the care provided throughout the perinatal period is vital for optimizing harm minimization through targeted education and primary health promotive strategies [30,39-41]. As highlighted previously, complementary research is currently underway focused on addressing educational preferences and follow-up needs of women after HDP [17]. To our knowledge, this is the first implementation of a co-designed targeted post-HDP education and follow-up package in Australia aimed at addressing knowledge gaps and enhancing capacity among HCPs to support care after HDP.

A recent publication by Slater et al [42] provides evidence to support our multidisciplinary co-design approach for targeted interventions to improve the uptake of evidence-based care after HDP. It also demonstrates demand for this research in the context of improving primary care management post HDP more broadly. Additionally, although not HDP-specific, MacKay et al [43] developed a complex health system intervention for improving systems of care in the context of hyperglycemia. MacKay et al [43] used implementation frameworks to support sustainable implementation in the Northern Territory and Far North Queensland. This research further supports our methodological approach to implementing a multifaceted post-HDP education and follow-up package in Australia’s complex health system.

Current Australian postnatal transitions of care following pregnancy are inadequate, highlighting the lack of appropriate handover between services as a barrier to the uptake of recommended follow-up care [18]. This research is therefore timely in the context of the integrated care landscape in Australia.

### Strengths and Limitations

The research aims to bridge an identified practice gap by enhancing knowledge and improving health care systems to promote uptake of contemporary evidence-based care guidelines. Adopting a collaborative and evidence-informed approach in consultation with health care providers (the end-users) is a key strength of the study. Although the focus on metropolitan Sydney may limit the generalizability, the multisite design will provide valuable context-specific insights for future scale-up.

The planned sample size of GPs (n=10) and HCPs (n=5‐10) is appropriate for the pilot study design. However, the small sample size limits statistical power and generalizability. Furthermore, the targeted recruitment of ANSC GPs likely favors early adopters. However, the complexities of health system design provide challenges for implementing meaningful system change. Therefore, meaningful and sustained engagement is essential for providing insight to inform evidence-based strategies for future scale-up.

Furthermore, the study period may limit the ability to influence permanent digital health solutions (follow-up intervention) due to lengthy approval processes. However, the use of evidence-based methodological implementation and reporting frameworks will promote replicable and robust results to influence policy and practice.

### Conclusions

This pilot implementation study will determine whether a post-HDP education and follow-up service package can be designed, developed, and implemented among GPs and maternity hospitals across Sydney. The findings of this research will provide an opportunity to influence contemporary development of effective post-HDP communication strategies, thereby improving the transition of care between tertiary and primary care services, and informing future implementation at scale.

## Abbreviations

ANSC: antenatal shared care
CESPHN: Central Eastern Sydney Primary Health Network
GP: general practitioner
HCP: health care provider
HDP: hypertensive disorders of pregnancy
REDCap: Research Electronic Data Capture
StaRI: Standards for Reporting Implementation Studies
TIDieR: Template for Intervention Description and Replication
